# Combined Statistical Analysis of Glioblastoma Outcomes—A Neurosurgical Single-Institution Retrospective Study

**DOI:** 10.3390/medicina60081234

**Published:** 2024-07-30

**Authors:** Ligia Gabriela Tataranu, Georgiana Adeline Staicu, Anica Dricu, Serban Turliuc, Dan Paunescu, Amira Kamel, Radu Eugen Rizea

**Affiliations:** 1 Neurosurgical Department, University of Medicine and Pharmacy “Carol Davila”, 020022 Bucharest, Romania; rizea.radu.eugen@gmail.com; 2 Neurosurgical Department, Clinical Emergency Hospital “Bagdasar-Arseni”, 041915 Bucharest, Romania; cdpaunescu@yahoo.com (D.P.); kamel.amyra@yahoo.com (A.K.); 3 Biochemistry Department, University of Medicine and Pharmacy, 200349 Craiova, Romania; adstaicu@gmail.com (G.A.S.); anica.dricu@live.co.uk (A.D.); 4 Medical Department, University of Medicine and Pharmacy “G. T. Popa”, 700115 Iasi, Romania; serban_turliuc@yahoo.com

**Keywords:** glioblastoma, neurosurgery, outcomes, survival rate, brain malignancy

## Abstract

*Background and Objectives*: Notwithstanding the major progress in the management of cancerous diseases in the last few decades, glioblastoma (GBM) remains the most aggressive brain malignancy, with a dismal prognosis, mainly due to treatment resistance and tumoral recurrence. In order to diagnose this disease and establish the optimal therapeutic approach to it, a standard tissue biopsy or a liquid biopsy can be performed, although the latter is currently less common. To date, both tissue and liquid biopsy have yielded numerous biomarkers that predict the evolution and response to treatment in GBM. However, despite all such efforts, GBM has the shortest recorded survival rates of all the primary brain malignancies. *Materials and Methods*: We retrospectively reviewed patients with a confirmed histopathological diagnosis of glioblastoma between June 2011 and June 2023. All the patients were treated in the Third Neurosurgical Department of the Clinical Emergency Hospital “Bagdasar-Arseni” in Bucharest, and their outcomes were analyzed and presented accordingly. *Results*: Out of 518 patients in our study, 222 (42.8%) were women and 296 (57.14%) were men. The most common clinical manifestations were headaches and limb paralysis, while the most frequent tumor locations were the frontal and temporal lobes. The survival rates were prolonged in patients younger than 60 years of age, in patients with gross total tumoral resection and less than 30% tumoral necrosis, as well as in those who underwent adjuvant radiotherapy. *Conclusions*: Despite significant advancements in relation to cancer diseases, GBM is still a field of great interest for research and in great need of new therapeutic approaches. Although the multimodal therapeutic approach can improve the prognosis, the survival rates are still short and the recurrences are constant.

## 1. Introduction

Glioblastoma (GBM) represents the most aggressive brain malignancy, and despite major recent advances in the management of cancerous diseases, it still has a dismal prognosis and the lowest reported survival rates [1,2,3]. Patients with GBM have poor outcomes firstly due to resistance to the available therapeutic options and secondly due to tumoral recurrence after neurosurgical excision [1,4]. In order to obtain a diagnosis, neuroimaging techniques, especially magnetic resonance imaging with gadolinium-based contrast agents, and biopsies are performed. However, the diagnosis is confirmed based on the histopathological and molecular results obtained after performing a tissue and/or liquid biopsy [5,6]. These molecular and histopathological results represent key factors in the current management of the disease, given that many established and potential biomarkers can predict the prognosis, response to treatment, recurrence, and survival in patients with GBM [7]. Currently, patients with GBM can benefit from a multimodal therapeutic approach based on neurosurgical excision, chemotherapy, especially with temozolomide, and radiotherapy. Research has demonstrated that combining these options leads to better outcomes [8].

## 2. Materials and Methods

This study aimed to evaluate the outcomes of patients presenting with GBM after being treated in the Third Neurosurgical Department of the Clinical Emergency Hospital “Bagdasar-Arseni”, Bucharest, Romania. The inclusion criteria were as follows: (1) patients over 18 years old with histopathologically confirmed GBM; (2) patients with cerebral involvement only; and (3) patients who provided their informed consent to take part in retrospective clinical studies. The exclusion criteria were as follows: (1) patients surgically treated in other departments who eventually attended with tumoral recurrences; and (2) patients with coexistent malignancies.

To select the aforementioned patients, we interrogated the hospital’s databases for the terms “glioblastoma”, “malignant glioma”, and “malignant brain tumor” between June 2011 and June 2023. After finding all of the patients with these diagnoses, we selected only those treated in our department. Subsequently, we evaluated the physical file of each patient, first of all, to verify if they provided a signature of approval or disapproval to take part in research studies and to confirm the diagnosis. Although many patients were great candidates for our retrospective analysis, they did not approve of taking part in such studies; thus, we excluded them. Finally, we performed a thorough appraisal of the patients’ data, reviewing elements such as clinical notes, demographics, histology, neuroimaging features, clinicopathological data, neurosurgical treatment, and outcomes.

### Statistics and Replicability

The current retrospective study was the result of reviewing the medical records from our institutional database of 518 patients treated for GBM in our department between June 2011 and June 2023. We extracted and evaluated patient demographics, clinicopathological features, clinical course, therapeutic options, and outcomes from the medical records. We determined the correlations between the acquired data to draw conclusions. Statistical analyses of the experimental data, tables, and figures were carried out using GraphPad Prism version 10.2.1 (339) software, as well as well as Microsoft Excel 16.87 software. The Chi-square test (Fisher’s exact test, χ^2^) was applied to evaluate the differences in variables and correlate normally distributed data, while *p*-values less than 0.05 were recorded as statistically significant. The overall survival was calculated from the date of histopathological diagnosis to the date of death, and the method of choice to estimate the survival rate was the Kaplan–Meier method (the log-rank Mantel–Cox test, Gehan–Breslow–Wilcoxon test, and Mantel–Haenszel test were used to compare the survival curves).

## 3. Results 

### 3.1. Demographic Profile, Clinicopathological Characteristics, and Correlation Analysis

The total number of selected patients who met the inclusion requirements for the study was 518 (n = 518), of whom 222 (42.85%) were women and 296 (57.14%) were men. The median age at GBM diagnosis was 62 years (32–84) for women and 59 for men (24–88), while the mean age at GBM diagnosis was 60.89 years for women and 58.04 years for men. For the entire group, both women and men, the median age at GBM diagnosis was 60 years (24–88), while the mean was 59.26 years (Table 1). Table 2 summarizes the general characteristics of the study group. 

**Figure 1:**
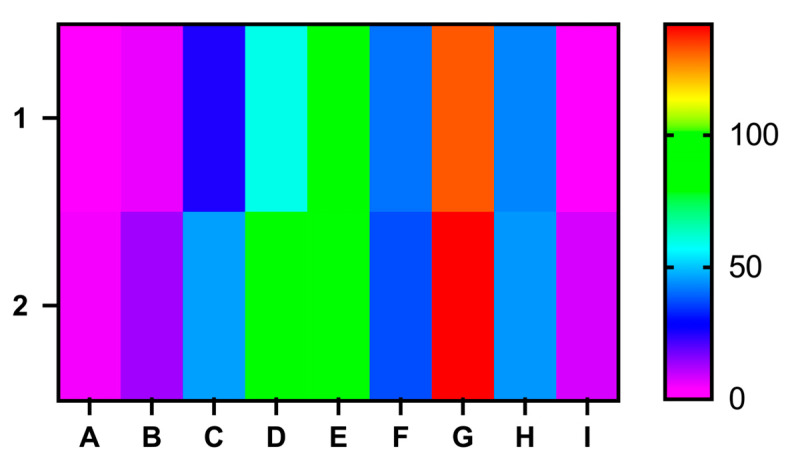
Heat map correlating the age groups at diagnosis of GBM in our study group with gender. 1—women; 2—men; age groups: A: 20–29 years; B: 30–39 years; C: 40–49 years; D: 50–59 years; E: 60–69 years; F: 70–79 years; groups G, H, and I summarized the groups of patients with an age equal to or above 60, 70, or 80 years old: G: >=60 years; H: >=70 years; I: >=70 years.

When split into age groups, a predominance of patients in the 60–69-year-old group was observed for both genders, with a total of 187 patients, of whom 89 (47.59%) were females and 98 (52.40%) were males (Figure 1). In the 20–29-year-old group, there were no females and only five males. In the 30–39-year-old group, comprising a total of 19 patients, 6 (31.57%) were women and 13 (68.42%) were men. The 40–49-year-old group included 71 patients, with 25 (35.21%) women and 46 (64.78%) men. The 50–59-year-old group had 59 (39.86%) women and 89 (60.13%) men, totaling 148. As we already mentioned, the 60–69-year-old group comprised the most patients. Lastly, the 70–79-year-old group had a total of 78 patients, of whom 41 (52.56%) were women and 37 (47.43%) were men. Furthermore, we selected another three groups for analysis: patients aged >=60 years, patients aged >=70 years, and patients aged >=80 years. In the >=60-year-old group, 132 (48%) patients were female and 143 (52%) were male, for a total of 275. In the >=70 years group, 43 (48.86%) patients were female and 45 (51.13%) were male, for a total of 88, while in the >=80 years group, there were two (20%) women and eight (80%) men, for a total of 10 patients. Furthermore, we found that 132 women and 143 men had GBM when they were older than 60 years old, while 90 women and 153 men had GBM when they were younger than 60 years old. This difference was statistically significant (OR 0.6373, RR 0.8259, 95% CI 0.4483–0.9059, *p* = 0.0119, χ^2^ = 6.331, df = 1). 

**Figure 2:**
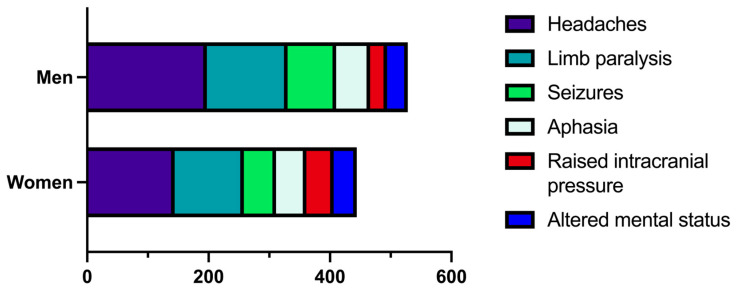
Bar graph describing the clinical symptoms at admission, as distributed by gender.

When admitted to our hospital, some patients had multiple symptoms, while others presented only one symptom. The most common clinical symptoms at admission were headaches (65.83%, n = 341), limb paralysis (47.68%, n = 247), and seizures (25.67%, n = 133), while aphasia (20.46%, n = 106), raised intracranial pressure syndrome (14.09%, n = 73), and altered mental status (13.89%, n = 72) were less frequent. When distributed by gender, we identified a richer symptomatology in men (Figure 2).

**Figure 3:**
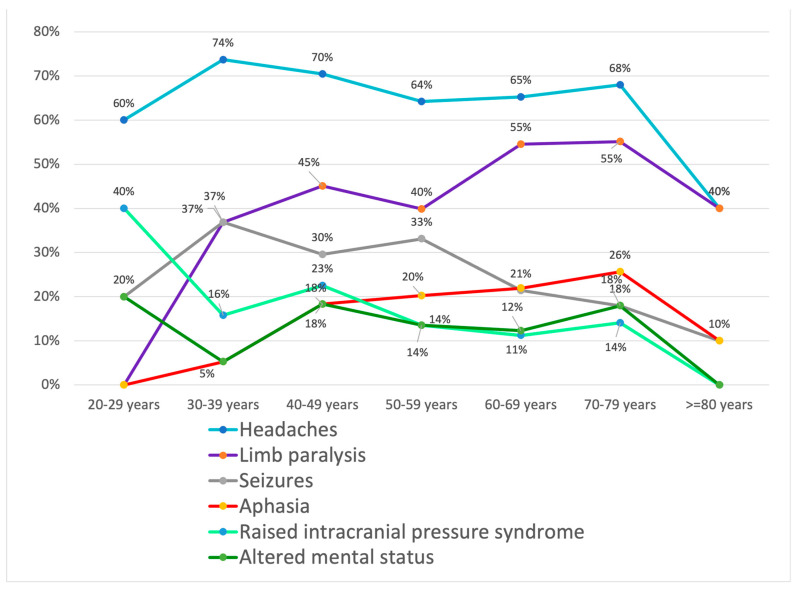
Clinical symptoms at admission, as distributed by age group.

In our study group, 144 (27.79%) women presented with headaches, 114 (22.00%) had limb paralysis, 53 (10.23%) had seizures, 50 (9.65%) had aphasia, 45 (8.68%) had raised intracranial pressure syndrome, and 38 (7.33%) had an altered mental status. In the group of men, the numbers were higher in relation to almost all the symptoms, as 197 (38.03%) of them presented with headaches, 133 (25.67%) with limb paralysis, 80 (15.44%) with seizures, 56 (10.81%) with aphasia, 28 (5.40%) with raised intracranial pressure syndrome, and 34 (6.56%) with altered mental status. When grouped by age group, we concluded that patients aged 80 years or older were the least symptomatic of all, while patients in the age group of 60–69 years were the most symptomatic (Figure 3).

In the 20–29-year-old group, three patients presented with headaches, none of them had limb paralysis, one had seizures, none of them had aphasia, two had raised intracranial pressure syndrome, and one had altered mental status. In the 30–39-year-old group, 14 patients presented with headaches, 7 with limb paralysis, 7 with seizures, 1 with aphasia, 3 with raised intracranial pressure syndrome, and 1 with altered mental status. In the 40- to 49-year-old group, 50 patients were admitted with headaches, 32 had limb paralysis, 21 had seizures, 13 had aphasia, 16 had raised intracranial pressure syndrome, and 13 had altered mental status. In the 50–59-year-old group, 95 patients presented with headaches, 59 had limb paralysis, 49 had seizures, 30 had aphasia, 20 had increased intracranial pressure syndrome, and 20 had an altered mental status. In the 60- to 69-year-old group, 122 patients were admitted with headaches, 102 had limb paralysis, 40 had seizures, 41 had aphasia, 21 had raised intracranial pressure syndrome, and 23 presented with altered mental status. In the 70- to 80-year-old group, 53 patients presented with headaches, 43 with limb paralysis, 14 with seizures, 20 with aphasia, 11 with raised intracranial pressure syndrome, and 14 with altered mental status. The group over 80 years old did not record symptoms such as altered mental status and raised intracranial pressure syndrome. However, four patients presented with headaches, four with limb paralysis, one patient had seizures, and one had aphasia.

**Figure 4:**
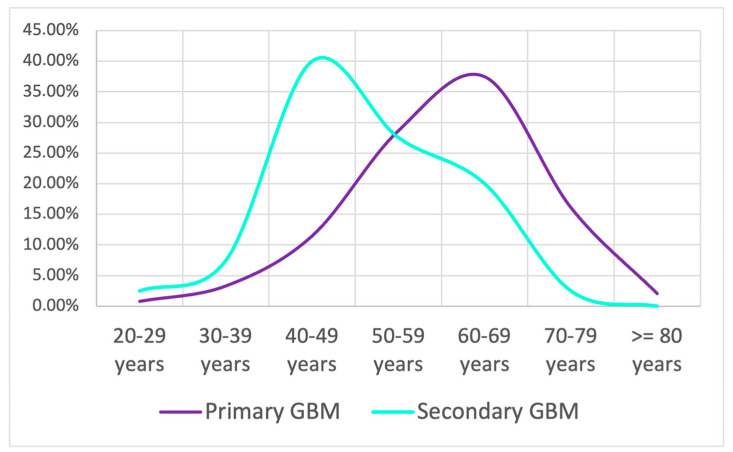
Age distribution of primary and secondary GBM in our study group.

Before 2021, GBM was classified according to the 2016 World Health Organization Classification of Central Nervous System Tumors, which was still based mainly on histological criteria. The terms “primary” and “secondary” were used to describe two epigenetically and genetically distinct entities [9,10]. Primary GBM used to be the most common and developed in patients with no prior record of brain tumors, with EGFR overexpression, PTN (MMC I) mutation, and CDKN2A (p16) deletion. Secondary GBM evolved from tumors of low grades and very often showed TP53 mutations [9,10,11,12]. In the current study, we used these terms, given that most of our patients were diagnosed before the new 2021 WHO CNS5 classification and were registered as such. Thus, in our study group, 478 (92.27%) patients had primary GBM, while 40 (7.72%) had secondary GBM. When distributed by age groups, the peak of primary GBM was recorded in the 60–69-year-old group, while the peak of secondary GBM was recorded in the 40–49-year-old group (Figure 4). 

**Figure 5:**
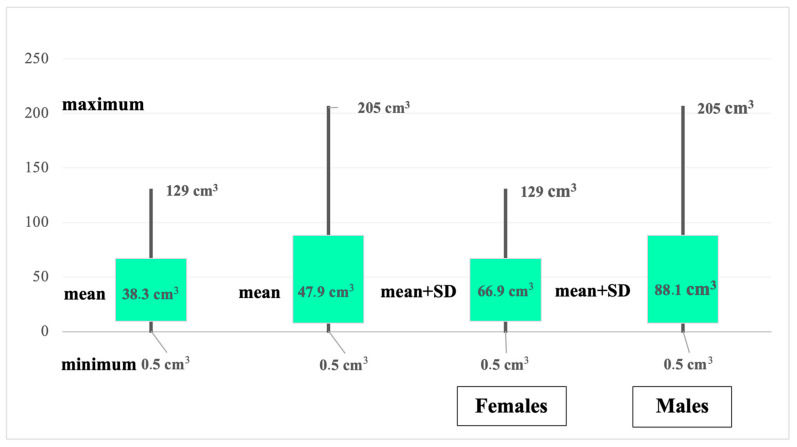
Tumoral volume distributed by gender.

Furthermore, we created two separate age groups that included patients aged 60 years old or younger (the first group) and patients aged 60 years old or older (the second group). There were 212 patients in the first group (40.92%) with primary GBM and 31 patients (5.98%) with secondary GBM. In the second group, there were 266 patients (51.35%) with primary GBM and 9 patients (1.73%) with secondary GBM (OR = 4.3218, RR = 1.1087, 95% CI 2.0136–9.2760, *p* = 0.0002, χ^2^ = 13.8; df = 1). Regarding the localization of the GBM, the most frequent single site was the frontal lobe (28.37%, n = 147), followed by the temporal lobe (15.63%, n = 81), and the parietal lobe (13.12%, n = 68). The least common location was in the occipital lobe (2.31%, n = 12). Cerebellar involvement was recorded in nine patients (1.73%). As for multiple lobes localization, the involvement of two lobes (32.23%, n = 167) was more frequent than the three lobes (6.56%, n = 34) localization. After analyzing the tumoral volume in our study group, we identified a great variability, with a minimum of 0.5 cubic centimeters (cm^3^) and a maximum of 205.41 cm^3^. Men showed a greater tumor volume when analyzed by gender. In females, the minimum tumoral volume was 0.5 cm^3^ and the maximum was 129 cm^3^ (mean = 38.3; SD = 28.6; mean-SD 9.7; mean + SD 66.9), whereas in males, the minimum tumoral volume was 0.5 cm^3^ and the maximum was 205.41 cm^3^ (mean = 47.9; standard deviation = 40.2; mean-SD 7.8; mean + SD 88.1) (Figure 5).

**Figure 6:**
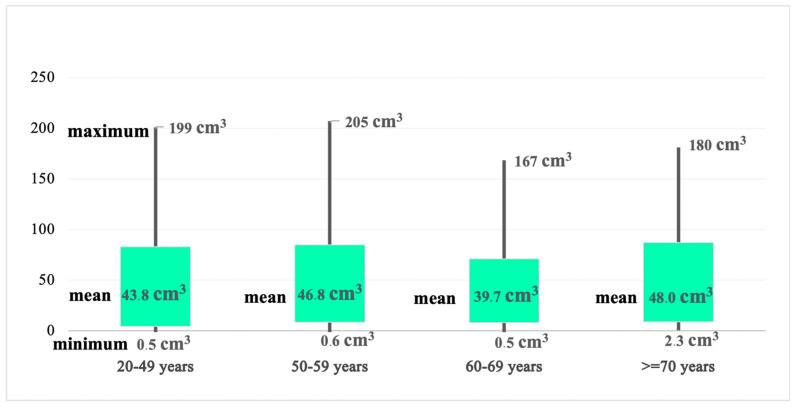
Tumoral volume distributed by age group.

In a similar manner, when distributed by age group, the biggest tumoral volumes were observed in patients aged 50–59 years, followed by patients aged 20–49 years and patients aged >=70 years, while the 60–69-year-old group had the smallest recorded tumoral volume (Figure 6).

### 3.2. Neurosurgical Results

**Figure 7:**
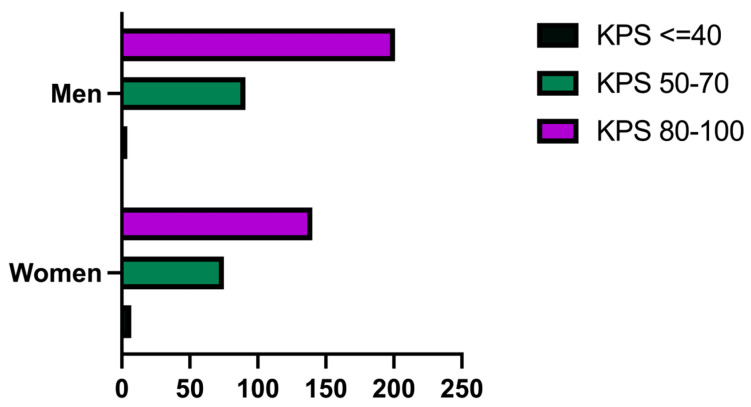
Bar graph describing the KPS at admission in patients with GBM in our study group, as arranged by gender.

The Karnofsky Performance Status (KPS) was recorded at admission and the results were divided into three groups. The first group included patients with a KPS equal to or less than 40, the second group included patients with s KPS between 50 and 70, and the last group included patients with a KPS between 80 and 100 (Figure 7). The first group comprised 11 patients, of whom 7 (63.63%) were women and 4 (36.36%) were men. The second group comprised 166 patients, of whom 75 (45.18%) were women and 91 (54.81%) were men. The third group comprised 341 patients, of whom 140 (41.05%) were women and 201 (58.94%) were men.

**Figure 8:**
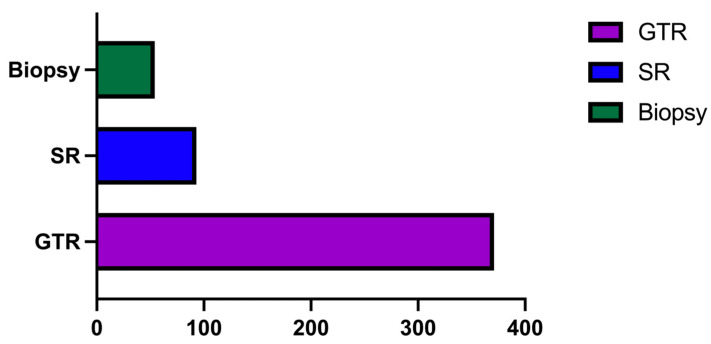
Bar graph describing the grade of tumoral resection in our study group.

Out of the 518 patients in our study group, in 371 (71.62%) cases, a gross total resection (GTR) was performed, in 93 (17.95%) cases, a subtotal resection (SR) was performed, and in 54 (10.42%) patients, a biopsy was performed (Figure 8).

**Figure 9:**
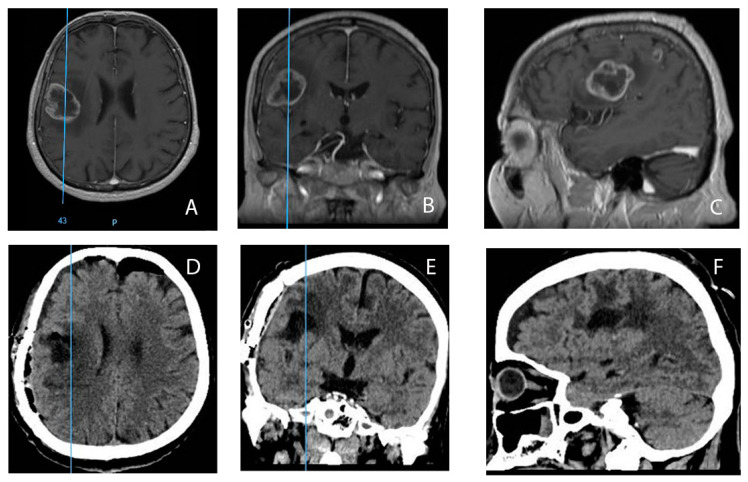
(**A**–**C**)—Preoperative brain MRI in a patient with GBM from our study group: contrast enhancement on T1-weighted axial MRI (**A**), coronal plane (**B**), and sagittal plane (**C**); (**D**–**F**)—Postoperative cerebral CT scan of the same patient after GTR: (**D**)—axial plane, (**E**)—coronal plane, and (**F**)—sagittal plane.

Figure 9 presents significant imaging details of a patient with GBM from our study group.

**Figure 10:**
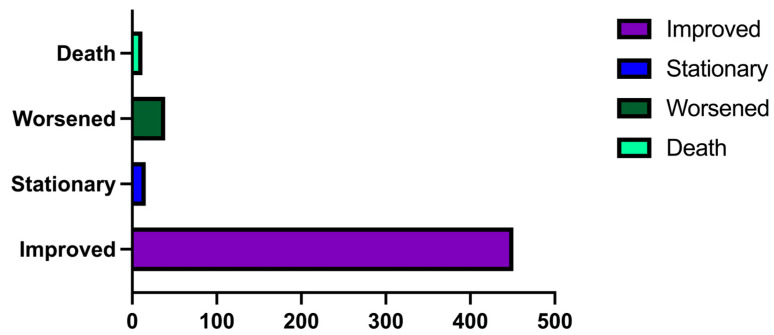
Bar graph describing the postoperative neurological results in our study group.

When it comes to the postoperative settings, 451 (87.06%) patients improved, 16 (3.08%) were stationary, 39 (7.52%) worsened, and 12 (2.31%) died (Figure 10).

The postoperative complications in our study group were also noteworthy. Hemorrhage-related complications (4.24%, n = 22) were the most common, followed by surgical wound-related complications (3.66%, n = 19) and death (2.31%, n = 12). Seizures (0.96%, n = 5) and hydrocephalus (0.77%, n = 4) were the least common postoperative complications. Out of 61 patients with diabetes mellitus, 15 (24.59%) had surgical wound-related complications, which means that out of 19 patients with surgical wound-related complications, 15 (78.94%) had diabetes mellitus. In a similar vein, out of 199 patients with cardiovascular comorbidities, 18 (9.04%) had hemorrhagic complications, which means that out of 22 patients with hemorrhage-related complications, 18 (81.81%) had cardiovascular comorbidities.

### 3.3. Survival Analysis

**Figure 11:**
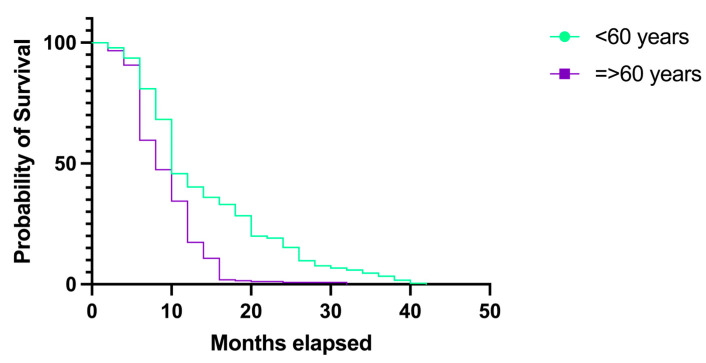
Kaplan–Meier plot describing the survival proportion in patients with GBM, as distributed by age groups.

We compared the survival rates in two patient groups based on age. The first group comprised patients younger than 60 years, while the second group included individuals aged 60 years or older. The probability of survival was higher in younger patients, and the overall survival was extended in this group as well. The median survival rate in patients younger than 60 years was 10 months (HR 1.25, 95% CI 1.050–1.489) in comparison to patients aged 60 years or older, which was 8 months (HR 0.80, 95% CI 0.671–0.952), *p* < 0.0001. In younger patients, the 12-month overall survival was 23.16%, while in older patients, it was 17.95%. The overall survival rate at 24 months was 9.45% in patients younger than 60 years and 0.57% in older patients (Figure 11). Only one patient survived 42 months, and this patient was in the young age group.

**Figure 12:**
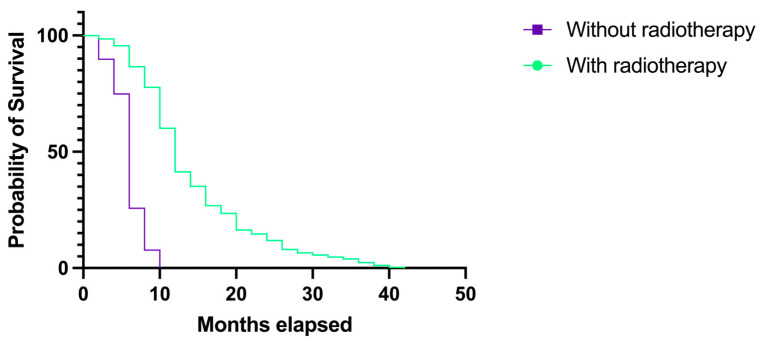
Kaplan–Meier plot describing the survival proportion in patients with GBM who underwent adjuvant radiation therapy versus patients who did not.

In our study group, a total of 339 (65.44%) patients underwent postoperative radiation therapy, while 179 (34.55%) did not. When analyzing the survival rates in these categories, we observed that patients who underwent radiotherapy had a longer median survival, and the difference was statistically significant. The median survival rate for patients who received adjuvant radiation therapy after surgery was 12 months (HR 2.00, 95% CI 1.661–2.408), but the median survival rate for patients who did not receive radiotherapy was only 6 months (HR 0.50, 95% CI 0.415–0.602), which is a 50% drop; *p* < 0.0001 (Figure 12).

**Figure 13:**
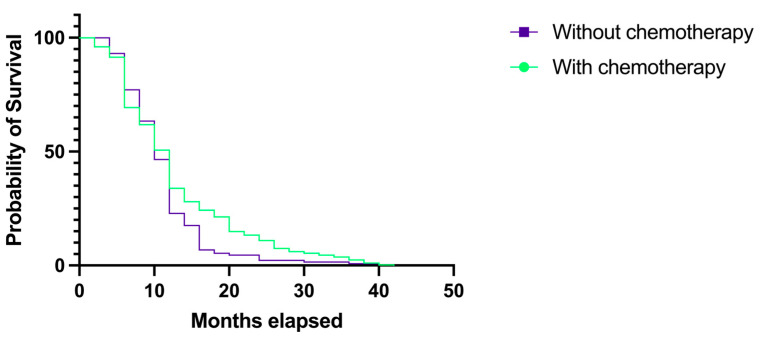
Kaplan–Meier plot describing the survival proportion in patients with GBM treated with concomitant and adjuvant chemotherapy.

In terms of chemotherapy after the neurosurgical intervention, 375 (72.39%) patients underwent concomitant and adjuvant chemotherapy (mainly with temozolomide), which prolonged their survival rates (Figure 13). The patients treated with chemotherapy had a median overall survival rate of 12 months (HR 1.20, 95% CI 0.983–1.464). The median survival rate for the remaining 143 (27.60%) patients, who did not receive chemotherapy, was 10 months (HR 0.83, 95% CI 0.683–1.017). However, the difference between the survival rates was not statistically significant (*p* = 0.2092).

**Figure 14:**
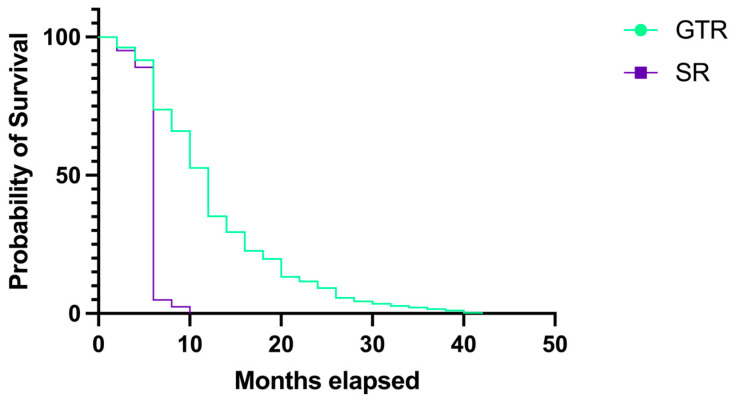
Kaplan–Meier plot describing the survival proportion in patients with GBM in whom we performed a GTR and a SR.

The patients for whom we performed a GTR had a greater probability of survival and a longer overall survival. Figure 14 shows that the difference in survival rates between the two groups was statistically significant (*p* < 0.0001). The survival rate in the first group was 12 months (HR 0.34, 95% CI 0.240–0.492), while the survival rate in the SR group was 6 months (HR 2.90, 95% CI 2.030–4.167). 

**Figure 15:**
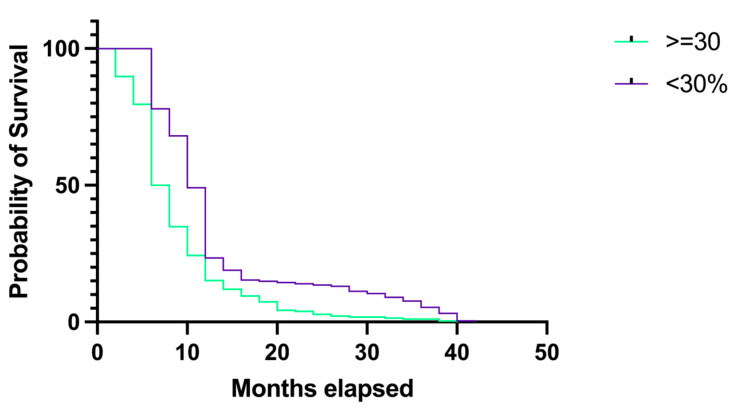
Kaplan–Meier plot describing the survival proportion in patients with GBM when distributed by tumoral necrosis grade.

To assess the survival rates of GBM patients when distributed by tumoral necrosis grade, we created two groups. The first group included patients with less than 30% necrosis, while the second group included patients with a percentage equal to 30% or greater. The first group comprised 222 (42.85%) patients, and the second comprised 296 (57.14%) patients. The median survival rate in the first group was 10 months (HR 1.42, 95% CI 1.199–1.703), while in the second group it was 7 months (HR 0.70, 95% CI 0.587–0.834), and the difference was statistically significant; *p* < 0.0001 (Figure 15).

## 4. Discussion

Glioblastoma is the most frequently encountered primary brain malignancy in adults, accounting for approximately 48% of cases, with a highly aggressive behavior and a propensity for recurrence [13]. Despite significant research in the last few decades, the treatment for GBM remains the same. The standard of care comprises maximally safe surgical resection, followed by radiotherapy plus concomitant and adjuvant temozolomide (Stupp regimen). Even with such a multimodal approach, the prognosis is still poor [13]. However, there have been big steps forward in terms of technology in the fields of surgery and biomolecular sciences. Safe maximal tumor resection is now easier to perform, and targeted therapeutic agents are showing promise [1,14]. 

In this study, we analyzed 518 adult patients diagnosed with GBM and treated in our neurosurgical department in the Clinical Emergency Hospital “Bagdasar-Arseni”, Bucharest. We observed a higher incidence of GBM development in men (296 patients, 57.14%) compared to women (222 patients, 42.85%) across all the age groups, except for the 70–79-year-old group, which consisted of 41 (52.56%) women and 37 (47.43%) men (a total of 78 patients). Moreover, when it comes to age, the peak incidence of GBM diagnosis was 59.26 years for the entire group, while the mean age at GBM diagnosis was 60.89 years for women and 58.04 years for men.

In a recent article written by Grochans et al. on GBM, the authors stated that age is a major factor in the development of GBM, and the age at diagnosis is usually equal to or greater than 65 years, followed by the group of age between 41 and 60. Furthermore, the authors concluded that the incidence of GBM increases with age, peaking at 75–84 years and decreasing after 85 years. In addition, when it comes to gender and its role in this disease, the study identified a higher incidence of GBM in male patients, citing the protective role of sex hormones in women [15].

Similarly, Kim et al. stated that GBM is more likely to be associated with older ages, citing not only the pro-inflammatory processes that are enhanced in advanced ages but also the upregulation of immunosuppressive factors that can impair antitumor immune system function. Moreover, the study revealed that another cause that sustains the age–GBM relationship is the susceptibility to local injuries in the aged brain from reactive oxygen species, as well as stalled or impaired blood flow in some cerebral areas. However, these are just a few reasons that could explain the age-dependent relationship, and further studies are needed in order to discover the full explanation [16].

Lin et al. established an age group classification for risk stratification and concluded that this classification is effective in assessing the risk of GBM. Hence, the authors concluded that the most appropriate classifications would be as follows: 0–14 years for the pediatric group, 15–47 years old for the young patients group, 48–63 years old for the middle age group, and equal to or greater than 65 year old for the elderly group. The authors stated that this classification can be used for preliminary assessment and has a major role in performing precise management of the disease. Furthermore, specific molecular ad histological differences were observed in every age group [17].

Thus, all of our results regarding age–gender relationships are consistent with similar prior studies.

Patients younger than 60 years not only had a longer probability of survival but also experienced an extended overall survival. One patient with GBM survived for 42 months. The median survival in this group was 10 months in comparison to the group of patients aged 60 years or older, in which it was 8 months, and the difference was statistically significant (*p* < 0.0001). Scholars have long debated the role of age as a prognostic factor in GBM, concluding that older ages may be associated with a poorer prognosis. For example, in 2020, Lin et al. concluded in a study that GBM and a large tumor size increase with age, and these conclusions were statistically significant (*p* = 0.000; *p* = 0.018). The same study suggested that GBM accounts for most of the adult population from the study group, with the greatest percentage in the ≥64-year-old group (66.3%), followed by the 48–64 group (46.2%), and 15–48-year-old group (22.9%) [17]. In a similar manner, Ohgaki et al. preformed a study that comprised five age groups and concluded that the overall survival in older patients was significantly shorter than in younger patients (4.1 months versus 8.8 months) [18]. Another relevant recent study was conducted by Jia et al., in which the authors concluded that there is a correlation between age and GBM, as younger patients had longer survival rates, while older patients had shorter survival rates [19]. Moreover, even older studies demonstrated relevant results. Smith et al. demonstrated that in patients with GBM, univariate analysis of nine variables found that the survival rates were associated with the patients’ age, and this was statistically significant (*p* < 0.001) [20]. Similarly, Burger et al. reaffirmed the strong negative relationship between survival rates after neurosurgical intervention and advancing age [21].

When it comes to the association between age at GBM diagnosis and sex, the statistical analysis (OR 0.6373, RR 0.8259, 95% CI 0.4483–0.9059, *p* = 0.0119, χ^2^ = 6.331, df = 1) concluded that the risk of developing GBM as a man is 0.6 times higher. Similar results were obtained by Thakkar et al. The authors concluded that GBM is more frequent in men and individuals of the white race and non-Hispanic ethnicity, and the mean age at diagnosis was 64 years [22]. However, in our study, all of the patients were of the white race. Consistent with these results, Aldape et al. showed that men are affected more commonly than women by a ratio of 1.6:1, and age is strongly associated with survival [23].

Our current study not only concluded that men have a tendency for GBM but also that this category has a richer symptomatology. Men were more likely to present at admission with headaches (38.03% versus 27.79% in women), limb paralysis (25.67% versus 22.00%), seizures (15.44% versus 10.23%), and aphasia (10.81% versus 9.65%), while women were more likely to present with raised intracranial pressure (8.68% versus 5.40% in men) and altered mental status (7.33% versus 6.56% in men). Similar correlations were described by Yang et al. in a study from 2019. The authors identified sex differences in the frequency of GBM development (more frequent in men) and the appearance and variety of symptoms [24]. Later, Carrano et al. described sex-specific differences in GBM, finding a protective role of estrogen and the upregulation of androgen receptors and testosterone as having detrimental effects in patients with this disease [25].

Before 2021, we used to classify GBM into primary and secondary [26], which are now defunct terms. We diagnosed approximately 80% of our patients with primary or secondary GBM because they received treatment prior to the World Health Organization’s (WHO) 2021 Classification of Central Nervous System Tumors, Fifth Edition (CNS5). As a result, we included correlations from this approach in the statistical analysis. According to the old classification, approximately 90% of GBMs will arise in patients with no prior history of clinical or histological cerebral lesions, and this type is called primary or de novo GBM [9,27]. On the other hand, 10% of GBMs progress from a less malignant precursor, a condition known as secondary GBM [9,27]. In line with these findings, the majority of patients in our study group (92.27%, n = 478) had primary GBM, while only a small group (7.72%, n = 40) received a secondary GBM diagnosis. The 60–69-year-old group recorded the peak of primary GBM, while the 40–49-year-old group recorded the peak of secondary GBM. Among the patients aged between 20 and 29 years, 0.48% had primary GBM, and 2.50% had secondary GBM. The 30–39-year-old group recorded 3.35% primary GBM and 7.50% secondary GBM. In the 40–49-year-old group, only 11.51% of the patients had primary GBM, in comparison to 40.00% who had secondary GBM. In the 50–59-year-old group, 28.66% of patients had primary GBM and 27.50% had secondary GBM, while in the 60–69-year-old group, 37.45% had primary GBM and 20.00% had secondary GBM. The study diagnosed 16.11% of patients aged 70 to 79 years with primary GBM and 2.50% with secondary GBM. In patients aged 80 years or older, the percentage of primary GBM was 2.09%, with no secondary GBM recorded in this group. 

When separated into patients aged 60 years or younger and patients aged 60 years or older, we concluded that older patients were more likely to develop primary GBM and younger patients were more likely to develop secondary GBM, and this correlation was statistically significant (*p* = 0.0002). The statistical analysis also showed that an older patient is four times more likely to develop a primary GBM than a secondary GBM (OR = 4.3218, RR = 1.1087, 95% CI 2.0136–9.2760, *p* = 0.0002, χ^2^ = 13.8; df = 1).

Currently, according to the WHO CNS5, the diagnosis of GBM is based mostly on biomolecular analysis rather than histopathology, as many researched biomarkers have demonstrated the ability to predict the disease prognosis and recurrence [28]. Therefore, GBM is defined as an IDH-wildtype adult-type diffuse glioma that exhibits necrosis and microvascular proliferation, with EGFR amplification, mutation of TERT promoter, or gain of chromosome 7 (+7) and loss of chromosome 10 (−10), and the new term is “GBM IDH-wildtype CNS WHO grade 4” [29]. Thus, primary GBM becomes GBM IDH-wildtype CNS WHO grade 4, and secondary GBM becomes astrocytoma IDH-mutant CNS WHO grade 4 [28]. 

However, regardless of its type, in our study group, GBM was mostly located in the frontal and temporal lobes as a single location and in two lobes more frequently as a multiple localization, and these findings were similar to those of other authors. Larjavaara et al. published a study regarding the incidence of gliomas by anatomic location. The study concluded that the most frequent subtypes were represented by GBM and the most common locations were the frontal (40%) and temporal (29%) lobes, followed by the parietal (14%) and occipital (3%) lobes. These differences in distribution remained even after adjustment for their tissue volume [30]. Correspondingly, Grech et al. concluded that the frontal lobe was the most frequent tumoral site in patients with GBM in their study, as identified in 25% (N = 25 patients) [31]. Furthermore, Liu et al. stated that older ages, larger tumoral sizes (cm), a frontal lobe location, and overlapping lesions were associated with GBM-related death, while a GTR, RT, and chemotherapy were protective factors. The same study suggested that the frontal lobe localization of GBM could negatively impact the prognosis, given its infiltrative nature, as well as its critical anatomical position [32]. Moreover, patients with frontal and temporal lobes localization are more prone to preoperative seizures in comparison to patients who have other GBM anatomic localization [33].

Analyzing the tumoral dimensions once again confirmed the male patients’ propensity for GBM. The minimum tumoral volume was 0.5 cubic centimeters (cm^3^), and the maximum was 205.41 cm^3^. However, the men’s volumes were higher than the women’s (min. 0.5 cm^3^; max. 205.41 cm^3^ versus min. 0.5 cm^3^; max. 129 cm^3^ in women). Similarly, patients aged 50–59 years recorded the largest tumoral volumes, while those aged 60–69 years recorded the smallest. In the 20–49-year-old group, the minimum tumoral volume was 0.5 cm^3^ and the maximum was 199 cm^3^ (mean 43.8 cm^3^; SD 38.9; mean-SD 4.76; mean + SD 82.7). In the 50–59-year-old group, the minimum tumoral volume was 0.6 cm^3^ and the maximum was 205 cm^3^ (mean 46.8 cm^3^; SD 37.9; mean-SD 8.89; mean + SD 84.72). The minimum tumoral volume in 60–69-year-olds was 0.5 cm^3^, and the maximum was 167 cm^3^ (mean 39.7 cm^3^; SD 31.2; mean-SD 8.43; mean + SD 70.95). In the >=70-year-old group, the maximum tumoral volume was 180 cm^3^ and the minimum was 2.3 cm^3^ (mean 48.0 cm^3^; SD 38.8; mean-SD 9.14; mean + SD 86.88). However, Zhiying Lin et al. stated that the tumoral size of GBM may increase with age (4–6 cm in older patients), and this correlation was statistically significant (*p* = 0.018). Furthermore, the study concluded that the tumoral size might impair the accuracy of the relationship between the survival rate and age [17].

The KPS at admission in our department was less than 40 in 11 (2.12%) patients, was between 50 and 70 in 166 (32.04%) patients, and was between 80 and 100 in 341 (64.83%) patients. Generally, poorer KPSs were observed in women, while higher KPSs were observed in men. Many authors stated that the KPS can reflect the prognosis of the postoperative course of the disease, and this theory is consistent with our results, given the fact that most of our patients with lower scores had a worse prognosis. For example, Gunawan et al. showed in their study from 2020 that good initial KPSs are up to five times more likely to have better outcomes at the 2-month follow-up after neurosurgical intervention. The median KPS before the neurosurgical intervention was 50 and 60 at 2 months after the surgery, with a favorable outcome KPS in 63.8% [34].

Chambless et al. stated that in patients with GBM, the postoperative KPS score has superior predictive value when compared to the preoperative score, and it has a superior ability to predict survival rates. Furthermore, the authors suggested that the preoperative KPS should be replaced by the postoperative one when estimating the prognosis [35].

However, Bartz et al. concluded that patients with a preoperative KPS score equal to or greater than 60% might benefit from surgical reduction of the tumor burden. The median preoperative score was 60%, while the postoperative score was 50%. Of all the patients who have undergone neurosurgical tumoral resection, 22.67% improved their KPS at discharge, 33.3% remained the same, and 44.0% worsened. The authors stated that there was no difference between patients who received surgical resection and patients who received biopsy only [36].

Regarding the neurosurgical intervention, the most important purpose was to perform a maximally safe resection to minimize the risk of future recurrence. Moreover, we aimed to reduce the overall intracranial mass effect for immediate neurological improvement, especially in patients with acute onsets. However, given the tumoral localization and infiltrative features of GBMs, in many cases, only a subtotal resection (SR) was possible. While the majority of patients (71.62%, n = 371) underwent a GTR, some patients (10.42%, n = 54) only underwent a biopsy. This choice might be explained by the fact that in some cases, a biopsy was needed to achieve a precise diagnosis, either because the patients were poor candidates for surgery or because they specifically opted for a biopsy in the first place while accepting the surgery afterward. Regarding the survival of patients and the extent of tumoral resection, our study concluded that patients in whom we performed a GTR had better survival rates when compared to patients with an SR, and these results were similar to those of other authors.

A relevant example is the study of Skardelly et al., which demonstrated a shortening of survival rates by a factor of 0.88 for a residual tumoral volume of 10 cm^3^. The authors not only suggested that there is a relationship between survival rates and the extent of tumoral resection but also that the preoperative tumor size can influence survival rates [37]. Similarly, Brown et al. published one of the most relevant studies regarding the matter. The authors demonstrated that in comparison to patients with an SR, patients with a GTR were 61% more likely to survive 1 year and 19% likely to survive 2 years. Furthermore, these patients were 51% more likely to remain progression-free at 12 months [38]. Consistent with these results, Youngblood et al. concluded, in a study from 2020 on the role of resection in the management of GBM, that the extent of the resection can influence the survival rates. The authors concluded that a greater extent of resection is correlated with increased survival rates [39]. Another recent study from last year performed by Karschnia et al. drew attention to the relevance of the extent of resection. Thus, the authors concluded that lower residual tumoral volumes were correlated with better outcomes, and extensive resection in non-contrast-enhanced tumors was associated with longer survival rates. It is worth mentioning that the prognostic value of tumoral resection classes in patients with GBM in this study was retained when adjusting for molecular and clinical markers [40].

However, Ringel et al. showed in their study that the age at the first neurosurgical intervention for GBM and the age at the re-resection influenced the survival rates, and that the extent of resection at initial surgery did not affect the survival rates and the time to re-resection [41].

In terms of the postoperative settings, 87.06% (n = 451) of patients improved, 3.08% (n = 16) were stationary, 7.52% (39) worsened, and 2.31% (12) died. The poor preoperative status, in which most patients arrived in a coma (Glasgow Coma Scale 3) due to acute onset or aggravation of symptoms, explains the number of deaths. However, none of these patients died intraoperatively, either due to poor surgical management or intraoperative surgical errors. The most frequent postoperative complications were represented by hemorrhage (4.24%, n = 22) and surgical wound-related complications (3.66%, n = 19), while the least common were seizures (0.96%, n = 5) and hydrocephalus (0.77%, n = 4). The risk of hemorrhages in GBM patients has been discussed before, although there is only limited data available, and it has been stated that out of all the cancerous diseases, GBM has one of the greatest risks of bleeding as well as thrombosis [42,43]. In our study group, wound-related complications were more common in patients with diabetes mellitus, while hemorrhages were more common in older patients with cardiovascular comorbidities.

Regarding the chemotherapeutical treatment, in our current study, 65.44% (n = 339) of patients underwent postoperative radiation therapy and showed a longer probability of survival and overall survival, while 34.55% (179) were not treated with postoperative radiotherapy and showed a shorter survival rate that was reduced by half, while the difference was statistically significant, with *p* < 0.0001 (median survival rate of 12 months with radiotherapy versus 6 months without radiotherapy). Similarly, patients treated with postoperative chemotherapy had longer survival rates, but the correlation was not statistically significant (*p* = 0.2092).

Witthayanuwat et al. showed that treatment with chemotherapeutic agents in patients with GBM can positively impact the outcomes. The authors showed that patients with postoperative chemotherapy and radiotherapy had longer survival rates, and they concluded that a multimodal approach is the best management option [44].

In a like manner, Perry et al. obtained similar results while analyzing the benefits of a multimodal approach in the management of patients with GBM. The study concluded that in elderly patients with GBM, the addition of chemotherapeutic agents (temozolomide) and radiotherapy prolonged the survival rate in comparison to radiotherapy alone (9.3 months versus 7.6 months median) and progression-free survival (5.3 months versus 3.9 months median). Furthermore, patients with methylated MGMT had even better results when treated with both chemotherapy and radiotherapy in comparison to radiotherapy alone (13.5 months versus 7.7 months median) [45].

The importance of the resection grade in GBM patients was highlighted in our study by the longer survival rates in patients with a GTR (median survival rate of 12 months versus 6 months in SR, *p* < 0.0001). The last registered survival in an SR was at 10 months (0.38%), while the last registered survival in a GTR was at 42 months (0.19%). In the GTR group, survival at 6 months was 65.44%, at 10 months it was 16.21%, at 12 months it was 9.45%, and at the end of the period it was 0.19%.

When comparing the survival rates based on the tumoral necrosis grade, we found that patients with less than 30% tumoral necrosis had longer survival rates than patients with 30% or more tumoral necrosis (median survival rate of 10 months versus 7 months), and this difference was statistically significant (*p* < 0.0001).

One of the main limitations of this study is represented by the fact that approximately 80% of our patients were diagnosed with GBM before 2021. However, we are looking forward to elaborating on more studies based on the biomolecular diagnosis of GBM according to the WHO CNS5. Overall, consistent with prior findings, this current retrospective study showed that a multimodal therapeutic approach may be beneficial for GBM patients, and neurosurgery can be the mainstay of treatment, specifically for patients with acute neurological aggravation due to mass effect. We are looking forward to more studies regarding this issue, which will possibly include patients treated with specific new targeted immunotherapies, as the new biomolecular advancement will bring more promising results.

## 5. Conclusions

Despite important medical progress in relation to cancer diseases, the glioblastoma research field is still developing, and patients still have a dismal prognosis with short survival rates. This study summarizes the outcomes of glioblastoma patients, concluding that a multimodal approach can be beneficial and can almost double the survival rates. In line with previous research, we affirm that men are more likely to develop GBM, given their higher diagnosis rates, richer symptomatology, and larger tumoral volumes. Neurosurgical treatment is associated with a low risk of complications, and the chances of increasing survival rates are higher if a gross total resection of the tumor is performed.

## Figures and Tables

**Table 1:** Age–gender analysis in our study group.

	Women	Men	Total
Number (%)	222 (42.85%)	296 (57.14%)	518 (100%)
Mean age at diagnosis (years)	60.89	58.04	59.26
Minimum age (years)	32.00	24.00	24.00
Maximum age (years)	84.00	88.00	88.00
Standard deviation (SD)	9.70	11.41	10.79
Median age at diagnosis (years)	62.00	59.00	60.00

**Table 2:** General characteristics of patients with GBM in our study group.

Total Number of Patients n =		518	Percent
Gender	Female	222	42.86%
	Male	296	57.14%
Clinical symptoms at admission	Headaches	341	65.83%
	Limb paralysis	247	47.68%
	Seizures	133	25.68%
	Aphasia	106	20.46%
	Raised intracranial pressure syndrome	73	14.09%
	Altered mental status (Glasgow Coma Score less than 15)	72	13.90%
Diabetes mellitus	Yes	61	11.78%
	No	457	88.22%
Cardiovascular comorbidities	Yes	199	38.42%
	No	319	61.58%
GBM localization	Frontal lobe	147	28.38%
	Temporal lobe	81	15.64%
	Parietal lobe	68	13.13%
	Occipital lobe	12	2.32%
	Cerebellum	9	1.74%
	Two lobes	167	32.24%
	Three lobes	34	6.56%
Tumoral necrosis grade	<10%	48	9.27%
	10–29%	174	33.59%
	30–49%	133	25.68%
	50–69%	97	18.73%
	70–89%	52	10.04%
	>=90%	14	2.70%
Tumoral volume (cm^3^)	Minimum	0.50	0.10%
	Maximum	205.41	39.65%
	Mean	43.89	8.47%
	Standard deviation	36.02	6.95%
Karnofsky Performance Status (KPS) Scale at admission	<40	11	2.12%
	50–70	166	32.05%
	80–100	341	65.83%
Grade of tumoral resection	Gross total resection	371	71.62%
	Subtotal resection	93	17.95%
	Biopsy	54	10.42%
Postoperative adjuvant radiotherapy	Yes	339	65.44%
	No	179	34.56%
Postoperative adjuvant chemotherapy	Yes	375	72.39%
	No	143	27.61%
Postoperative complications	Yes	62	11.97%
	No	456	88.03%

## Data Availability

Datasets analyzed or generated during the study are unavailable due to privacy and ethical restrictions. This study has ethical permission (No. 24672/30 May 2024).
